# Systems-Level Integration of Multi-Omics Identifies Genetic Modifiers of TANGO2 Deficiency Disorder

**DOI:** 10.3390/biom15121742

**Published:** 2025-12-16

**Authors:** Manuel Airoldi, Heather Bondi, Veronica Remori, Silvia Carestiato, Giovanni Battista Ferrero, Alfredo Brusco, Mauro Fasano

**Affiliations:** 1 Department of Science and High Technology, University of Insubria, 22100 Como, Italy; mairoldi@uninsubria.it (M.A.); heather.bondi@uninsubria.it (H.B.); vremori@uninsubria.it (V.R.); 2 Department of Neuroscience “Rita Levi Montalcini”, University of Turin, 10124 Turin, Italy; silvia.carestiato@unito.it (S.C.); alfredo.brusco@unito.it (A.B.); 3 Medical Genetics Unit and Thalassemia Center, San Luigi University Hospital, 10049 Orbassano, Italy; giovannibattista.ferrero@unito.it; 4 Department of Clinical and Biological Sciences, University of Turin, 10124 Turin, Italy; 5 Medical Genetics Unit, Città della Salute e della Scienza University Hospital, 10126 Turin, Italy; 6 Neuroscience Research Center, University of Insubria, 21052 Busto Arsizio, Italy

**Keywords:** TANGO2 deficiency disorder, multi-omics, network medicine, genetic modifiers

## Abstract

TANGO2 deficiency disorder is a rare autosomal recessive disease (~100 cases reported worldwide). Despite being caused by loss-of-function variants in the *TANGO2* gene, patients exhibit marked phenotypic variability, including intrafamilial differences among individuals carrying identical variants. To uncover potential modifier mechanisms influencing disease severity, we developed an integrative Systems biology framework, combining exome sequencing, transcriptomics, variant effect prediction, and Human Phenotype Ontology mapping. This approach was applied to two siblings carrying identical compound heterozygous *TANGO2* variants but opposite clinical outcomes: one severely affected and one asymptomatic. Personalized protein–protein interaction networks and combined univariate and multivariate analyses were employed to maximize specificity in this single-family comparison. In the affected sibling, a cumulative burden of common *APOB* variants, together with altered *VLDLR*, *NTN1*, and *LDHA* expression, implicated disrupted lipid metabolism and neurodevelopmental pathways. The asymptomatic sibling harbored a potentially protective 3′-UTR variant in *EP300* and no *APOB* variant burden, supporting enhanced post-transcriptional regulation within developmental biology networks. These findings highlight lipid metabolism as a key pathway in TANGO2 deficiency pathophysiology and suggest autophagy and mitophagy as additional modifier mechanisms influencing phenotypic variability. Our integrative multi-omics framework provides a valuable strategy for elucidating genotype-phenotype relationships in rare diseases and supports personalized therapeutic approaches.

## 1. Introduction

Systems biology is an interdisciplinary approach that seeks to understand biological complexity by integrating diverse layers of molecular information, such as genomics, transcriptomics, proteomics, and metabolomics, within the framework of networks and dynamic systems [1]. Rather than examining single genes or molecules in isolation, it models how genes, proteins, and pathways interact to influence health and disease. This approach has greatly advanced the understanding of disease mechanisms, particularly through the construction of molecular interaction networks and the identification of key drivers of disease phenotypes, even in small or genetically heterogeneous cohorts [2,3]. By mapping protein–protein interactions (PPI) and signaling pathways, Systems biology provides a holistic view of cellular processes that is especially valuable for rare Mendelian disorders, which often remain poorly characterized due to limited patient numbers and scarce functional studies of disease-associated genes. Even when patients carry identical causative mutations, rare diseases frequently show marked phenotypic variability. In such cases, secondary variants may act as genetic modifiers (gene variants able to modify the phenotypic outcome of the primary disease gene) and thereby influence disease severity and contribute to clinical variability [4]. Identifying these genetic modifiers remains a major challenge, especially in the context of rare diseases where traditional association studies lack statistical power [4]. Advanced statistical and multivariate methods are therefore essential to extract mechanistic insight from high-dimensional multi-omics datasets, particularly in neurodevelopmental disorders and other rare conditions where sample sizes are limited but molecular complexity is high [5]. Network-based analyses have proven effective in pinpointing key regulatory nodes and functional modules that drive disease pathogenesis, which may serve as therapeutic targets [6]. Moreover, the integration of omics data with curated phenotype repositories, such as the Human Phenotype Ontology (HPO, a structured vocabulary of phenotypic abnormalities), facilitates mapping molecular signatures to clinical features, providing deeper insights into disease heterogeneity and underlying biological processes [7].

TANGO2 (Transport and Golgi Organization 2 homolog) deficiency disorder (TDD, OMIM: #616878) is a rare autosomal recessive disorder caused by pathogenic variants in the TANGO2 gene, first described in 2016 [8,9]. TDD presents a broad clinical spectrum, including neurodevelopmental delay, brain atrophy, dystonia, seizures, and hypothyroidism. Metabolic stresses, such as infections or prolonged fasting, can trigger life-threatening crises characterized by rhabdomyolysis, cardiac arrhythmias, and encephalopathy [10].

The *TANGO2* gene is evolutionarily conserved across mammals and encodes a ubiquitously expressed protein. Initially, it was believed to be localized in the cytosol and Golgi apparatus, playing a role in Golgi-to-endoplasmic reticulum (ER) membrane redistribution [9,11,12]. More recent studies suggest its localization within mitochondria [8,13,14,15,16]. Interestingly, TANGO2 also localizes to contact sites among the ER, lipid droplets (LDs), and mitochondria [15]. Proteomic analyses of TANGO2-deficient human fibroblasts revealed perturbations in crucial metabolic pathways, including fatty acid oxidation, ER-Golgi network, and secretory pathways [17]. TDD patient-derived cells show abnormal mitochondrial morphology, increased reactive oxygen species (ROS), altered lipid droplet structure, and disrupted lipid metabolism [15,18]. Lipidomic analysis of TANGO2-deficient cells revealed elevated free fatty acid pools, triglycerides and lysophospholipids, confirming a significant imbalance in the lipid profile [19]. Notably, purified TANGO2 binds acyl-coenzyme A (acyl-CoA), suggesting a role in lipid metabolism relevant to the metabolic crises observed in TDD [16].

While TDD shows complete penetrance, the symptom severity varies widely, even among siblings carrying the same *TANGO2* variants [20]. This phenotypic heterogeneity suggests that additional genetic interactions (e.g., genetic modifiers), epigenetic mechanisms, or environmental factors may influence clinical outcomes in TDD patients [4,18].

The aim of this work is to elucidate the molecular mechanisms underlying phenotypic variability in two siblings with identical heterozygous *TANGO2* variants (c.262C>T, p.Arg88Ter; c.338del, p.Gly113AlafsTer10), previously reported by Miyake et al. [20], that result in complete loss of TANGO2 protein expression. Despite sharing the same pathogenic variants, the siblings exhibit strikingly different clinical outcomes. The older brother (Subject 1) shows a severe TDD phenotype, with neurodevelopmental delay, motor and cognitive impairments, hypothyroidism, and multiple metabolic crises, including cardiac arrest. In contrast, his younger sister (Subject 2) remains entirely asymptomatic. To uncover genetic modifiers underlying this phenotypic variability, we applied an integrated Systems biology approach combining genomic, transcriptomic, and phenotype ontology. This robust framework allowed us to identify differences between the two siblings, even in the absence of suitable controls and despite the extreme rarity of the disease, which underlie the siblings’ differing clinical presentations, particularly the younger sister’s unusual resilience to TDD.

## 2. Materials and Methods

### 2.1. Patients and Genomic Analysis

Two siblings with TDD carrying the same compound heterozygous variants in the *TANGO2* gene (c.262C>T, p.(Arg88Ter); c.338del, p.(Gly113AlafsTer10)) were selected to be studied. Both variants are classified as pathogenic (PVS1, PM2). c.338del and c.262C>T variants were paternally and maternally inherited, respectively. Despite sharing identical genotypes, their clinical presentations were markedly different (Table 1) [20]. This unique intra-familial contrast provides a controlled framework to investigate genotype–phenotype correlations and potential molecular modifiers of disease severity.

Genomic DNA was extracted from peripheral white blood cells using the PureLink Genomic DNA kit (Thermo-Fisher Scientific, Waltham, MA, USA), and exomic regions were enriched with the Illumina Nextera^®^ Rapid Capture Exome Kit (Illumina, San Diego, CA, USA), with whole exome sequencing (WES) conducted at 80X mean coverage. Paired-end FASTQ files were aligned to the human reference genome (GRCh38/hg38) using the BWA-MEM algorithm. Variant calling format (VCF) files were processed from WES performed on the proband, following standard protocols outsourced to GalSeq. Variants in the *TANGO2* gene identified through WES were further confirmed by Sanger sequencing.

Variant annotation was performed using the Variant Effect Predictor (VEP, Ensembl Release 113, October 2024, https://www.ensembl.org/Tools/VEP, accessed on 2 November 2024), incorporating data from multiple sources, including gene identifiers, population allele frequency (Genome Aggregation Database, gnomAD ver.2.1), and functional predictions. Variant deleteriousness was assessed using the Combined Annotation Dependent Depletion (CADD) score metapredictor (v1.7) [21]. Focus was placed on variants in protein-coding genes, excluding records lacking chromosome annotations, gene symbols, or showing sequencing artifacts (e.g., ALT alleles > 25 bp). Only variants mapped to protein-coding or loss-of-function genes were retained. SNP and indel files were merged for each subject.

Subject-specific protein–protein interaction (PPI) networks were built in Cytoscape (version 3.10.3) using input gene lists from WES and retrieving experimentally validated physical interactions between proteins from the IMEx (International Molecular Exchange) consortium database [22]. A preliminary PPI network included first-degree interactors, refined by removing non-mutated interactors, self-loops, and duplicated edges. Only nodes corresponding to *Homo sapiens* (taxonomy ID: 9606) were retained.

Finally, variants were filtered based on allele frequency (AF < 0.01 or not annotated in gnomAD, as these criteria enrich for potentially rare or private variants), and the resulting physically connected genes were considered to functional enrichment analysis.

### 2.2. Total RNA Sequencing and Transcriptome Analysis

Skin fibroblasts from both subjects were cultured in T25 flasks using Dulbecco’s Modified Eagle Medium (DMEM, Gibco, Waltham, MA, USA), supplemented with 10% fetal bovine serum (FBS, Gibco, Waltham, MA, USA) and 1% Penicillin-Streptomycin (100×, Gibco). Cultures were maintained at 37 °C in a humidified atmosphere with 5% CO_2_. Cells were passaged every 4 days using 0.05% trypsin-EDTA (Gibco), and fibroblasts between passages 3 and 6 were used for RNA extraction.

Total RNA was isolated using TRIzol Reagent (Euroclone, Pero, Italy) and purified following the manufacturer’s instructions (Direct-zol RNA Miniprep Kit, Zymo Research, Irvine, CA, USA). RNA concentration and purity were assessed using a Nanodrop spectrophotometer (Thermo Fisher Scientific, Waltham, MA, USA) and an Agilent 2100 Bioanalyzer (Agilent Technologies, Santa Clara, CA, USA). RNAseq libraries were prepared using a strand-specific poly(A) selection protocol and sequenced on the DNBSEQ platform, generating 100 bp paired-end reads. Each sample yielded at least 20 million high-quality reads. Raw sequencing data (FASTQ files) were aligned to the human reference genome (GRCh38/hg38) using STAR 2.7.11a [23], and raw gene counts were obtained with the RSubread package (release 3.17) [24]. Count data were normalized to counts per million (CPM) and low-expression genes (CPM < 0.5 in at least two replicates) were filtered out [25]. Log-transformed CPM values were computed to identify potential discrepancies across replicates, and Principal Component Analysis (PCA) was performed to evaluate biological variance.

In accordance with recommendations for filtering polymorphic genes and homolog pairs, highly expressed and variable human leukocyte antigen (*HLA*) genes were removed, as their high polymorphism and variability could confound subsequent analyses.

Differential Gene Expression (DGE) analysis was performed to compare gene expression levels between Subject 1 and Subject 2 using both univariate and multivariate approaches. The univariate analysis was carried out using the DESeq2 package (version 1.42.0) [26], which models raw gene counts with normalization for library depth, providing a robust assessment of gene-level changes. Genes with an adjusted (Benjamini–Hochberg) *p*-value < 0.05 were considered differentially expressed (DEGs). No additional log_2_ fold-change cutoff was applied; however, log_2_-fold change values were computed and reported for interpretation. In contrast, for multivariate analysis, a Sparse Partial Least Squares Discriminant Analysis (sPLS-DA) combined with Recursive Feature Elimination (RFE) was employed [27], capturing coordinated expression patterns and identifying the most informative genes. The sPLS-DA method considers global expression patterns and identifies sets of genes that contribute most to subject discrimination. RFE was iteratively applied, reducing the feature set to the top 10% of genes based on predictive contribution. The 10% threshold was chosen to limit overfitting while retaining the most informative genes identified by the sPLS-DA model.

The final DEG list for downstream analysis was obtained by intersecting the results of DESeq2 and sPLS-DA/RFE, ensuring high confidence and reducing false positives. This integration of statistical and multivariate methods is particularly valuable in a 1 vs. 1 comparison, where the limited sample size makes specificity essential for biological interpretation. Differential expression was quantified as log2 fold change between Subject 1 and Subject 2.

Finally, the DEGs were used as input for functional interaction (FI) network construction in Cytoscape via ReactomeFIPlugIn [28] (version 8.0.9), which also added linker genes that functionally connect disconnected DEGs.

### 2.3. Topological Analysis of the Network

Topological analysis was performed on a directed network using the NetworkAnalyzer tool in the Cytoscape environment (version 3.10.3). The following topological parameters were measured for each node: edge count, in-degree, and out-degree. The edge count reflects the total number of connections associated with each node, while in-degree and out-degree quantify the number of incoming and outgoing edges, respectively. These metrics were used to assess node centrality and interaction flow, enabling the identification of hub genes and potential regulators within the network.

### 2.4. Functional Enrichment Analysis

Functional enrichment analysis was performed using WebGestalt 2024 (WEB-based Gene SeT AnaLysis Toolkit) [29] (both over-representation analysis—ORA—and gene set enrichment analysis—GSEA). The parameters were set as follows: Homo sapiens as organism of interest, Kyoto Encyclopedia of Genes and Genomes (KEGG) [30], Reactome [31], and Gene Ontology—cellular component [32] as databases. For statistical testing, the “genome protein coding” was used as reference set. To determine statistically significant enriched pathways a Benjamini–Hochberg false discovery rate (FDR) threshold of <0.05 was applied. Weighted set cover algorithm was applied to reduce redundancy in the results.

### 2.5. Phenotype Annotation and Omics Integration

The Human Phenotype Ontology (HPO) database (September 2024 release, https://hpo.jax.org) was queried using the term “TANGO2” to generate a virtual gene panel. This search identified the phenotype “Metabolic encephalomyopathic crises, recurrent, with rhabdomyolysis, cardiac arrhythmias, and neurodegeneration” (OMIM: #616878) as associated with TANGO2. We retrieved the list of genes annotated to each relevant HPO term. This gene set was used to build a disease-specific PPI network, where nodes are genes of the panel above and edges are the interactions between them. To integrate phenotype and functional data, we intersected this disease-phenotype network with the FI network, incorporating differentially expressed genes and their functional linkers relevant to the TDD phenotype.

All nodes were annotated with genomic variant data, including variant type, allele frequency, CADD score, and zygosity. To identify genes harboring variants with potential deleterious effects, a CADD score cutoff of ≥15 was arbitrarily defined, which corresponds to approximately the top 3.2% of the variants and represents the median score for canonical splice site and non-synonymous variants in CADD v1.0. This cutoff was chosen to capture variants with moderate to high predicted impact while maintaining a manageable set for downstream analysis. Genes harboring variants above this threshold were subjected to functional enrichment analysis. For identification of highly deleterious variants, we applied a more stringent cutoff of CADD score ≥ 30, corresponding to the top 0.1% of all possible variants. This more stringent cutoff focuses on variants most likely to have strong functional effects.

**Figure 1:**
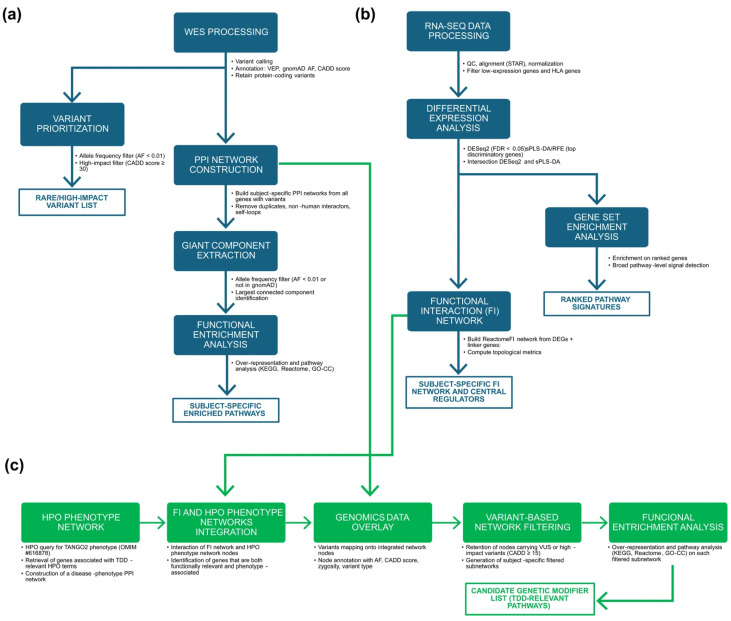
Overview of the multi-omics workflows used in this study. (**a**) Genomic data analysis workflow: Variant calling and annotation (VEP, gnomAD, CADD) were performed to characterize the variant set. Rare (AF < 0.01 or absent in gnomAD) or high-impact (CADD ≥ 30) variants were retained and used to construct subject-specific PPI networks based on IMEx interactions. After removal of non-human interactors, duplicates, and self-loops, the largest connected component of each network was extracted and analyzed through pathway over-representation analysis (KEGG, Reactome, GO-CC). (**b**) Transcriptomic data analysis workflow: RNA-seq data underwent quality control, alignment, normalization, and filtering of low-expression and HLA genes. Differential expression was assessed using DESeq2 (univariate) and sPLS-DA with recursive feature elimination (multivariate) to obtain a high-confidence set of DEGs. Pathway-level signals were evaluated through GSEA, and DEGs were used to reconstruct a ReactomeFI-based functional interaction (FI) network, expanded with linker genes and characterized through topological metrics. (**c**) Omics integration workflow: HPO terms associated with TDD (OMIM #616878) were used to build a disease–phenotype network, which was intersected with the FI network to identify genes that were both functionally connected and phenotype-associated. Subject-specific WES variants were overlaid onto this integrated network to obtain personalized integrated networks, which were then filtered using a CADD ≥ 15 threshold to generate subject-specific subnetworks. Functional enrichment analysis of these subnetworks was used to derive TDD-relevant functional signatures and to shortlist candidate genetic modifiers.

Figure 1 provides an overview of the analytical workflows employed in this study: (a) Genomic data analysis workflow (b) the Transcriptomic data analysis workflow, and (c) the integration workflow combining phenotype ontology, functional networks, and variant filtering to identify subject-specific candidate genetic modifiers.

## 3. Results

### 3.1. Genomic Data Analysis

**Figure 2:**
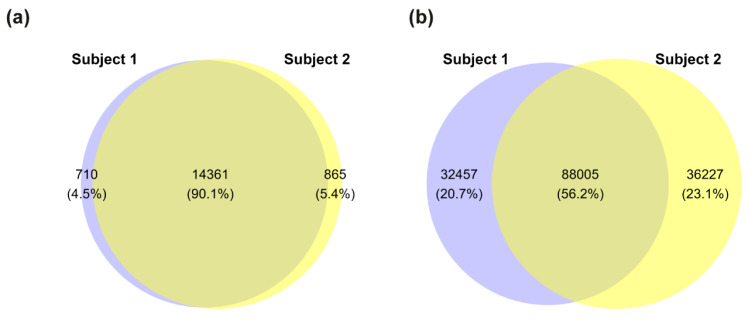
Distribution of genes and variants between siblings. (**a**) Euler-Venn diagram showing protein-coding genes with at least one detected variant in each subject. (**b**) Total number of genomic variants detected in each subject and their overlap. Blue = Subject 1 (male, affected), yellow = Subject 2 (female, unaffected).

As an initial step, we performed a comprehensive WES analysis to compare genomic profiles of the two siblings and identify variants that may contribute to their divergent clinical phenotypes. This analysis revealed variants in 15,071 protein-coding genes in Subject 1 (male) and 15,226 protein-coding genes in Subject 2 (female). Notably, 710 genes were unique to Subject 1 and 865 genes to Subject 2 (Figure 2a). In total, we identified 244,694 variants across both subjects: 120,462 in Subject 1 and 124,232 in Subject 2. Of these, 88,005 variants were shared, while 32,457 variants were unique to Subject 1 and 36,227 to Subject 2 (Figure 2b). The known compound heterozygous variants in the *TANGO2* gene (c.262C>T, p.Arg88Ter; c.338del, p.Gly113AlafsTer10) were confirmed in both siblings.

**Figure 3:**
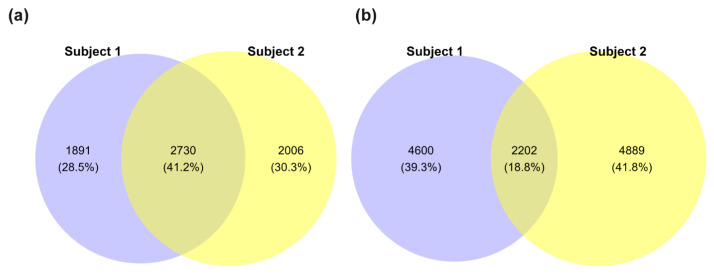
Rare variant distribution between siblings. (**a**) Euler-Venn diagram of protein-coding genes harboring at least one rare variant (allele frequency < 0.01 or not annotated in gnomAD). (**b**) Total number of rare variants detected in each subject and their overlap. Blue = Subject 1 (male, affected), yellow = Subject 2 (female, unaffected).

Next, we assessed the potential pathogenicity of the detected variants by comparing CADD score distributions between the siblings (Figure S1). However, no significant differences were observed. We then filtered for rare variants with an AF below 0.01 or lacking annotation in gnomAD. This filtering step reduced the number of protein-coding genes with variants to 4621 in Subject 1 and 4730 in Subject 2, with 2730 genes shared between them (Figure 3a). The total number of variants was also reduced, with Subject 1 carrying 6802 variants and Subject 2 having 4889, of which 2202 were shared (Figure 3b).

We focused on variants with a CADD score ≥ 30 to prioritize variants most likely to have strong deleterious effects, identifying a total of 48 rare variants (Table 2). Most of the variants identified (46) were located in genes that are tolerant to haploinsufficiency in heterozygosity, suggesting limited contribution to phenotypic divergence. We therefore prioritized variants located in genes with high intolerance to loss-of-function (pLI ≥ 0.9). Among these, a variant in *CUX1* was found in both siblings and thus unlikely to account for clinical differences. In Subject 1, the most pathogenic variant was found in the *PABPC1* gene, which encodes a poly(A) binding protein (CADD score: 45, AF: 1.824 × 10^−3^; pLI = 1). Notably, variants in this gene have been linked to neurodevelopmental disorders, potentially impairing neurogenesis [33,34]. Conversely, all high-CADD variants unique to Subject 2 (female, unaffected) were detected in genes not typically associated with TDD. These included a canonical splice-site variant in the *SORT1* (c.1108+1G>A) gene, classified as a variant of unknown significance (VUS). SORT1 is involved in glucose and lipoprotein metabolism, neurotrophin signaling and lysosomal targeting [35,36]. Additional missense variants were found in *KCNJ12*, *PAX7*, and *RANBP3* genes, which are mainly involved in cellular excitability, fetal development and cellular signaling, respectively [37,38,39]. However, these variants had allele count > 10 in gnomAD and lacked phenotypic relevance to Subject 2’s presentation and were therefore excluded from further consideration.

To explore the physical interactions among proteins harboring these variants, we constructed PPI networks. The initial networks comprised 15,071 nodes and 305,034 edges for Subject 1, and 15,226 nodes and 308,747 edges for Subject 2. After filtering for rare variants (AF < 0.01 or not annotated in gnomAD), Subject 1’s network was reduced to 4621 nodes and 33,996 edges, and Subject 2’s network to 4736 nodes and 35,604 edges. The giant components of these filtered networks contained 4071 nodes and 33,994 edges for Subject 1, and 4189 nodes and 35,601 edges for Subject 2 (Figure S2).

**Figure 4:**
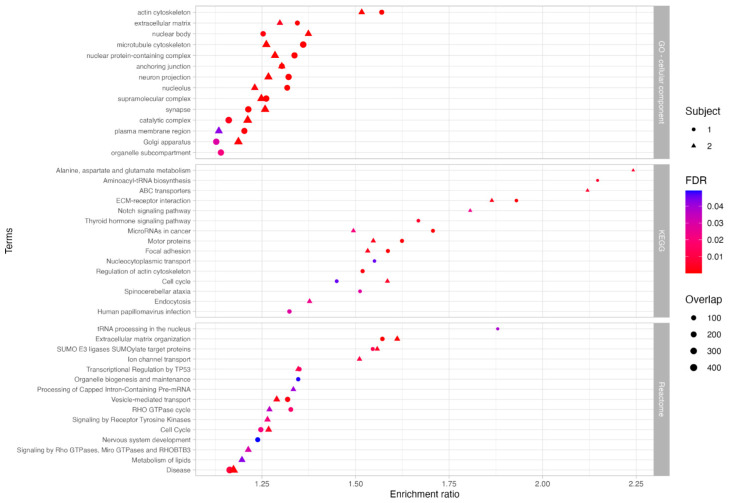
Functional enrichment analysis of genes in subject-specific PPI network giant components. Significantly enriched terms identified through functional enrichment using GO (Cellular Component), KEGG, and Reactome databases. Circles represent terms enriched in Subject 1; triangles represent terms enriched in Subject 2. Symbol size reflects the number of associated genes, and color indicates statistical significance (FDR ≤ 0.05).

To explore the potential biological significance of these variants, we performed functional enrichment analysis using ORA on the genes within the giant components. This analysis aimed to identify pathways potentially linked to the TDD phenotype and infer the subcellular localization of the encoded proteins (Figure 4, Table S1). In Subject 1, enriched pathways included thyroid hormone signaling, neurodevelopment, vesicle transport, cellular signaling, and transcriptional processes. In Subject 2, enriched pathways included amino acid and lipid metabolism, transport processes, cellular signaling, vesicular trafficking, motor protein function, RNA processing, extracellular matrix organization, endocytosis, and p53-related mechanisms. Gene Ontology (GO) analysis revealed enrichment in both Subjects for cellular components such as the cytoskeleton, extracellular matrix, plasma membrane, nucleus, neuronal projection, synapses, and Golgi apparatus. Notably, Subject 1 also showed specific enrichment in organelle subcompartments.

### 3.2. Transcriptomic Data Analysis

RNA sequencing analysis generated a raw count matrix of 31,638 genes across six samples (31,638 × 6). After filtering for low expression (CPM > 0.5 in at least two samples), the matrix was reduced to 16,489 × 6. Exploratory data analysis showed consistent raw count distributions across biological replicates, with the median log2CPM values for the entire dataset aligning with those of individual samples (Figure S3a). PCA further revealed distinct clustering of replicates by Subject, with the first two principal components explaining 68% and 15% of the variance, respectively (Figure S3b). Due to their intrinsic high variance, HLA genes were removed, resulting in a final 16,403 × 6 count matrix.

**Figure 5:**
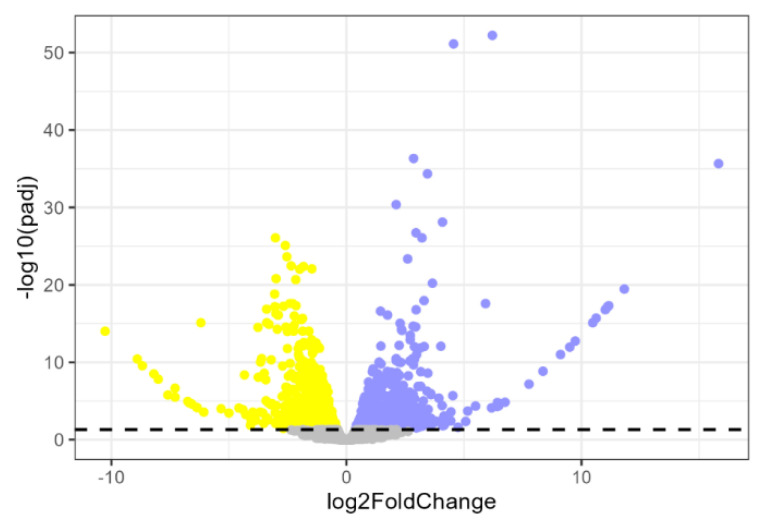
Differential gene expression between siblings. Volcano plot showing differentially expressed genes (DEGs) in Subject 1 relative to Subject 2. Blue dots represent up-regulated genes in Subject 1 vs. Subject 2; yellow dots represent down-regulated genes in Subject 1 vs. Subject 2; gray dots indicate non-significant genes. The dashed horizontal line marks the significance threshold (adjusted *p*-value = 0.05).

Differential expression analysis was performed using DESeq2, fitting the data to a negative binomial distribution (Figure S4). We identified 1425 differentially expressed genes (DEGs; adjusted *p*-value < 0.05). Of these, 673 were up-regulated and 752 were down-regulated genes in Subject 1 relative to Subject 2 (Figure 5).

To complement the univariate analysis, we applied a multivariate approach using sPLS-DA on the 16,403-gene matrix. After confirming data separability (Figure S5a), we selected the first component (explaining 31% of the variance) and iteratively performed RFE to identify the most discriminative genes. This process retained 1640 genes (10% of the initial genes), with the first component (X-variate 1) explaining 86% of the variance (Figure S5b).

**Figure 6:**
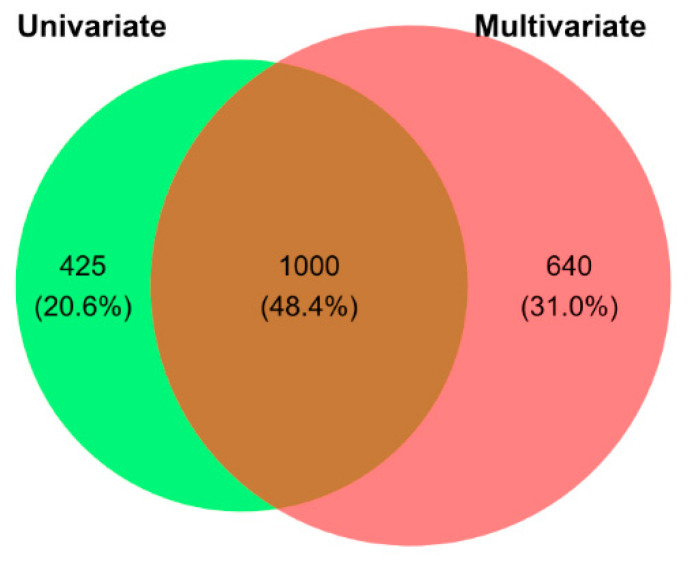
Overlap of differentially expressed genes (DEGs) identified by univariate and multivariate analyses. Venn diagram showing the overlap between DEGs identified using a univariate approach (green; DESeq2) and a multivariate approach (red; sPLS-DA with recursive feature elimination). The intersection includes 1000 DEGs retained for downstream analysis.

To ensure robustness and increase specificity, we intersected the DEGs identified by DESeq2 and sPLS-DA, retaining only the 1000 genes common to both methods (Figure 6). Among these, 454 were up-regulated and 546 down-regulated in Subject 1 vs. Subject 2 (Figure S6, Table S2).

GSEA, performed using the log2 fold changes across all genes, did not identify any significantly enriched pathways.

**Figure 7:**
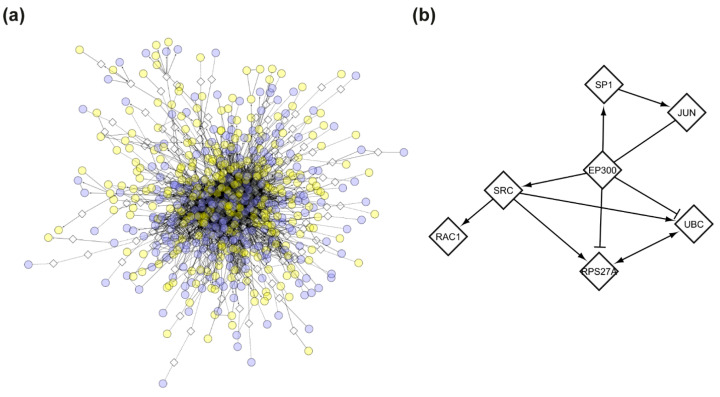
Functional interaction (FI) network of differentially expressed genes (DEGs). (**a**) Full FI network generated from DEGs with the addition of linker genes, showing functional connectivity among differentially expressed transcripts. (**b**) Core cluster of 7 highly connected genes within the network. Circular nodes represent DEGs; diamond-shaped nodes indicate linker genes. Edges represent high-confidence functional interactions; arrows indicate directed functional interactions; dashed edges indicate predicted (but not experimentally validated) functional interactions. Blue nodes are upregulated in Subject 1; yellow nodes are downregulated in Subject 1.

To further interpret the transcriptional changes, we constructed an FI network based on the DEG list. The FI network comprised 735 nodes (521 DEGs and 214 linker genes) and 3114 edges (Figure 7a). By focusing on high connectivity nodes (both in-degree and out-degree), we identified a tightly interconnected cluster of 7 genes at the network’s core (Figure 7b). Among these, *EP300* appeared to be the central player, with an in-degree of 24 and an out-degree of 91 (Table 3).

### 3.3. Integrating Genomic, Transcriptomic, and Phenotypic Data for Subject-Specific Networks

To integrate phenotypic information into the analysis, we queried the HPO database to identify genes associated with the TDD phenotype. Specifically, we selected the entry “Metabolic encephalomyopathic crises, recurrent, with rhabdomyolysis, cardiac arrhythmias, and neurodegeneration” (OMIM: #616878), which is representative of the clinical features observed in TDD. This entry was linked to 61 HPO terms associated with TDD (Table S3), corresponding to 4599 unique genes (Table S4). These genes were employed to generate a disease-phenotype network to capture functional relationships potentially relevant to the observed phenotype. The resulting network consisted of 4599 nodes and 50,016 edges. The giant component contained 4276 nodes and 50,015 edges, with the remainder comprising one node pair and 321 singleton nodes (Figure S7).

**Figure 8:**
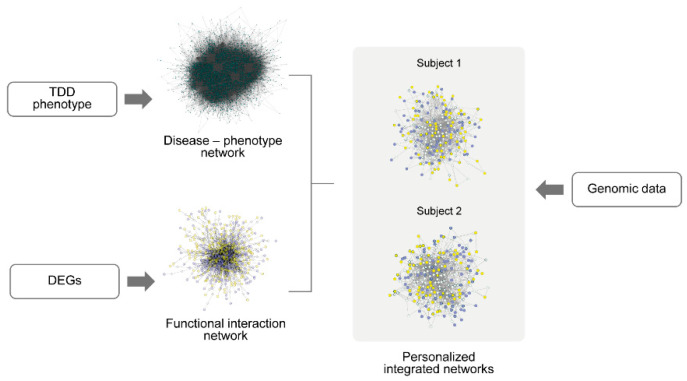
Workflow for the construction of personalized integrated networks. Differentially expressed genes (DEGs) were used to generate a functional interaction (FI) network, while phenotype-associated genes were retrieved from the Human Phenotype Ontology (HPO) database to construct a disease-phenotype network (OMIM: #616878). The giant components of both networks were intersected to define a shared functional-phenotypic network. Genomic variant data from WES were subsequently integrated to build subject-specific personalized networks.

To investigate how DEGs and their functional interactors relate to the TDD phenotype, we intersected the giant component of the disease-phenotype network with the previously constructed FI network based on DEGs. The overall integration workflow is illustrated in Figure 8.

**Figure 9:**
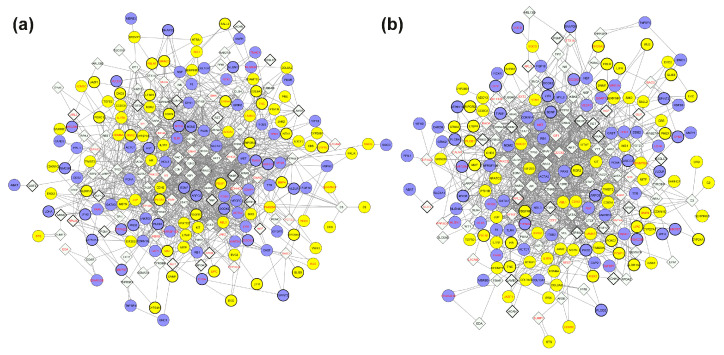
Personalized integrated networks combining exome sequencing, gene expression, and disease-phenotype data. (**a**) integrated Subject 1 network; (**b**) integrated Subject 2 network. Each network was generated by intersecting the giant component of the disease-phenotype network with the functional interaction network, followed by the integration of subject-specific exome sequencing data. Circles represent differentially expressed genes (DEGs), diamond-shaped nodes represent linker genes. Blue nodes indicate upregulated genes in Subject 1 (and correspondingly downregulated in Subject 2); yellow nodes represent downregulated genes in Subject 1 (and correspondingly upregulated in Subject 2). Gene symbols in red denote homozygous variants; gene symbols in black denote heterozygous variants.

Genomic data from WES were incorporated to enrich the network with subject-specific variant information, providing a more comprehensive molecular view. This integrative strategy resulted in the construction of subject-specific networks that integrate genomic, transcriptomic, and disease-phenotype information (Figure 9a,b).

**Figure 10:**
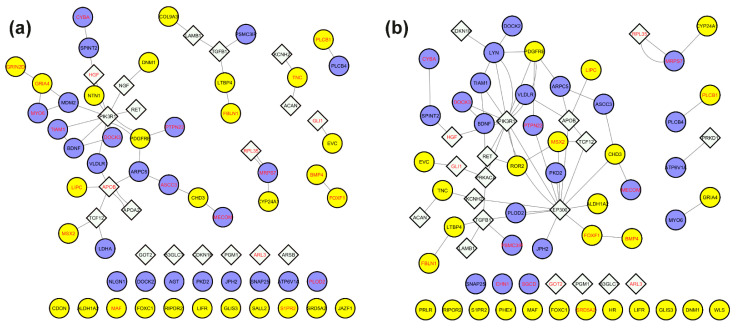
Subnetworks of genes carrying potentially impactful variants extracted from the personalized integrated networks. Nodes were filtered to retain only genes carrying variants classified as variants of uncertain significance (VUS) or pathogenic (CADD score ≥ 15). Panels (**a**) and (**b**) correspond to Subject 1 and Subject 2, respectively. Circular nodes indicate differentially expressed genes (DEGs); diamond-shaped nodes are linker genes. Gene symbols in red indicate homozygous variants; gene symbols in black indicate heterozygous variants. Blue nodes refer to upregulated genes in Subject 1 (and correspondingly downregulated in Subject 2); yellow nodes are downregulated genes in Subject 1 (and correspondingly upregulated in Subject 2).

To prioritize functionally relevant nodes, we filtered the subject-specific integrated networks by retaining only genes with variants exhibiting a CADD score ≥ 15 (Table S5). This filtering yielded a refined network of 71 nodes and 46 edges for Subject 1, consisting of a giant component (28 nodes, 33 edges), a secondary cluster (6 nodes, 5 edges), 2 triads, 3 node pairs, and 25 isolated nodes (Figure 10a, Table 4). For Subject 2, the filtered network contained 69 nodes and 64 edges, comprising a giant component with 41 nodes and 58 edges, 1 triad, 3 node pairs, and 19 isolated nodes (Figure 10b, Table 4).

For downstream analysis, we focused on the genes in the giant components of each subject-specific subnetwork. The genes and corresponding variants are listed in Table 4.

**Figure 11:**
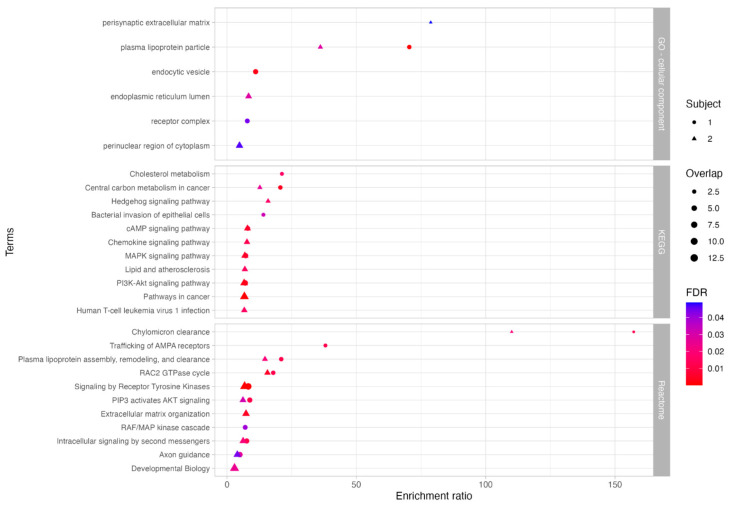
Functional enrichment analysis of genes carrying potentially impactful variants in the integrated networks. Genes in the giant components of the subject-specific integrated networks were filtered for variants classified as variants of uncertain significance (VUS) or pathogenic. Significantly enriched terms were identified using GO (Cellular Component), KEGG, and Reactome databases. Circles represent Subject 1; triangles represent Subject 2; symbol size corresponds to gene count, and color indicates statistical significance (FDR ≤ 0.05).

Functional enrichment analysis was then performed using the GO cellular component database to infer the possible cellular localizations of encoded proteins, and the KEGG and Reactome pathway databases to investigate significantly over-represented pathways (Figure 11, Table S6). Results revealed shared enrichment in lipoprotein particle localization and protein-lipid complexes in both subjects.

In Subject 1, we identified a heterozygous variant in *APOA2* (rs3813628) and, more strikingly, two homozygous variants in *APOB*: (c.8216C>T) and (c.1853C>T). While c.8216C>T (AF: 0.23; CADD: 27.5) is relatively common, it has been linked to elevated LDL cholesterol levels [40], increased cardiovascular risk [41], and altered statin response [42]. Similarly, c.1853C>T (AF: 0.401; CADD: 26.7) is also common, but has been associated with mild effects on lipid traits. However, this variant was present in Subject 2 in a heterozygous state, suggesting possible dosage effects.

Additionally, Subject 1 carried a rare 3′ untranslated region (3′ UTR) deletion in *NTN1* (c.30_33del); AF: 2.563 × 10^−4^; CADD score: 16.28), a gene crucial for axonal growth during neurogenesis [43]. Although 3′UTR variants are rarely pathogenic, this deletion could still affect regulatory elements involved in NTN1-mediated signaling, and subtle modulatory effects cannot be excluded. A unique pathway enriched only in Subject 1 was central carbon metabolism in cancer, which includes key metabolic processes involved in energy production and biosynthesis. Within this pathway, we detected a rare missense variant in *LDHA* (lactate dehydrogenase A; c.439G>T; AF: 1.256 × 10^−3^; CADD score: 23.8) in Subject 1, a critical enzyme in anaerobic glycolysis. This variant has been previously associated with elevated LDH serum levels and altered protein expression [44].

In Subject 2, we identified a 3′ UTR SNP in *EP300* (c.*13_*15del; AF: 0.245; CADD score: 15.8), a common variant previously associated with reduced schizophrenia risk [45]. Enrichment analysis also highlighted endoplasmic reticulum lumen localization, along with perturbations in lipid metabolism and cellular signaling, consistent with findings from Subject 1.

Only variants with the strongest biological rationale were highlighted; the others in Table 4 may still be relevant but currently lack interpretable evidence.

## 4. Discussion

TANGO2 deficiency disorder (TDD), a rare disease with an estimated prevalence of 1:1,000,000 [8,9,46], exhibits marked clinical variability even among individuals with identical pathogenic variants. This suggests that additional genetic factors modulate disease severity. We investigated two siblings with the same compound heterozygous pathogenic *TANGO2* variant but opposite phenotypes: Subject 1 was severely affected, while Subject 2 remained asymptomatic. This unique intrafamilial case provided an ideal model to investigate endogenous factors modulating disease severity while minimizing environmental and genetic background confounders, allowing a more precise identification of subject-specific molecular differences.

To systematically investigate the molecular basis of this phenotypic discordance, we employed an integrative Systems biology approach combining WES, transcriptomic profiling, phenotypic annotations, and functional network analysis. This strategy addressed the inherent limitations of single-case comparisons, such as the lack of external controls and other TDD-subjects. To overcome the statistical challenge, we integrated multivariate and univariate transcriptomic analysis, filtered variants by predicted pathogenicity and population frequency, and incorporated linker genes to build personalized networks. Notably, the consistency of the following results with current understanding of TDD suggested that this approach successfully mitigated the limitations inherent to single pair comparisons, extracting biologically meaningful information through convergent evidence across multiple molecular layers.

After excluding high impact variants (CADD ≥ 30) as primary drivers of the phenotypic discrepancy, we constructed a protein–protein interaction (PPI) network based on rare variants (GnomAD frequency ≤ 1%) for each subject. Functional enrichment analysis of the giant component of these two networks revealed distinct pathway perturbations. Subject 1 showed significant enrichment in the thyroid hormone signaling and nervous system development pathways, consistent with his severe clinical presentation. In contrast, Subject 2 showed enrichment in lipid metabolism, ABC (ATP-Binding Cassette) transporters, as well as alanine, aspartate, and glutamate metabolism pathways, corroborating the established role of *TANGO2* in lipid homeostasis [15] and supporting previous proteomic studies that reported alterations in amino acid metabolism in TDD patients [17]. These pathways are strongly linked to mitochondrial function and cellular energy homeostasis, as they provide substrates and reducing equivalents essential for ATP production through the TCA cycle and oxidative phosphorylation [47], which are crucial in preventing the metabolic decompensation observed in TDD patients. The observed enrichment of ABC transporters in Subject 2 may further enhance intracellular trafficking of lipids and metabolites, promoting metabolic flexibility under stress conditions [48]. Together, these mechanisms could provide a protective buffer against the energetic failures typically associated with TDD, partially contributing to the absence of clinical symptoms in Subject 2.

Transcriptomic profiling alone did not reveal any significantly altered pathways. Thus, we constructed a functional interaction (FI) network starting from the 1000 DEGs identified through a combined univariate and multivariate approach. To enhance biological interpretability and reveal potential regulatory mechanisms, we incorporated linker genes. Topological analysis of this network revealed a central cluster of seven highly connected genes, with *EP300* emerging as a key player (in-degree: 24, and out-degree: 91).

EP300 is an acetyl-CoA–dependent acetyltransferase known to suppress autophagy when cytosolic acetyl-CoA levels are high through the acetylation of key autophagy regulators [49,50]. Although TANGO2 binds long-chain acyl-CoAs rather than acetyl-CoA, acyl-CoA metabolism feeds into the acetyl-CoA pool via β-oxidation, suggesting that metabolic alterations relevant to TDD could plausibly influence EP300 activity and autophagy-linked regulatory pathways. Since TANGO2 is linked to mitochondrial function, and given that acetyl-CoA is a signaling metabolite to control mitophagy [51], it cannot be excluded that mitophagy may also represent a relevant pathway to explore in future studies aimed at understanding phenotypic variability in TANGO2-deficient patients.

*EP300*, which presented a deletion only for Subject 2 and was involved in the “Developmental Biology” pathway enriched in Subject 2, may play a crucial regulatory role contributing to her asymptomatic status. Notably, *EP300* and the other genes in the central hub were not differentially expressed themselves, but emerged as critical linkers that connect and potentially regulate multiple DEGs. The remaining six genes carried variants with low predicted functional impact (CADD < 15) and participated in diverse pathways, but no single pathway was shared by all seven genes; for this reason, they were not discussed individually. This finding highlights the added value of network-based inference in rare disease transcriptomics, where relevant biological signals may be distributed across weakly differentially expressed genes and become evident only when considered within a functional framework.

By integrating DEGs, linker genes implicated in the disease phenotype, and subject-specific genetic information, and then filtering for genes with CADD score ≥ 15, we identified 71 variants in Subject 1 and 69 variants in Subject 2, respectively. Functional enrichment consistently highlighted lipid-related pathways in both subjects, emphasizing the established role of TANGO2 in lipid homeostasis [52]. TANGO2 localizes near mitochondria-ER contact sites and lipid droplets, and its loss disrupts lipid composition and fatty acid metabolism [15]. This prompted us to examine specific genetic variants in lipid pathways that might underlie the phenotypic divergence [53], such as those in *APOB* and *VLDLR* genes.

Indeed, the homozygous co-occurrence of both *APOB* variants (c.8216C>T, p.Pro2739Leu) and (c.1853C>T, p.Ala618Val) in Subject 1 points to a possible additive impact. This configuration represents a cumulative burden of variants, whereby multiple missense changes may a combined influence. Although *APOB* is considered tolerant to loss-of-function variants (pLI: 0), the biallelic presence of these missense variants may still lead to a subtle impairment of APOB functions. This could contribute to intracellular lipid accumulation and trigger compensatory mechanisms, as evidenced by the upregulation of lipoprotein receptors like VLDLR [54] in Subject 1. Interestingly, while both siblings carried heterozygous *VLDLR* variants, only Subject 1 showed *VLDLR* upregulation, suggesting a specific regulatory response to a more pronounced lipid imbalance in the severely affected sibling. In contrast, Subject 2 carried *APOB* (c.1853C>T) only in heterozygosity, lacking the homozygous burden observed in Subject 1’s *APOB* gene. This difference in *APOB* genotype, leading to potentially less impaired APOB function, could mitigate the lipid dysregulation associated with TANGO2 deficiency, thereby contributing to Subject 2’s remarkable resilience. Since TANGO2 depletion itself is known to cause enlarged lipid droplets and dysregulated lipid handling, the presence of these potentially damaging variants in *APOB*, a key mediator of triglyceride mobilization from lipid droplets for lipoprotein assembly [55], together with differential *VLDLR* expression, may critically exacerbate the lipid imbalance in Subject 1.

Beyond lipid metabolism, other pathways also offer insights into the siblings’ phenotypic differences. Axon guidance, a pathway significantly enriched in Subject 1, directly correlates with his prominent neurological involvement, including cognitive and motor impairments. Within this pathway, we identified the variant (c.*30_*33del) in *NTN1* gene, which encodes a secreted protein crucial for axonal pathfinding during neuronal development. Although this variant does not alter the coding sequence, it may affect the post-transcriptional regulation, a hypothesis supported by the observed downregulation of *NTN1* in Subject 1 compared to his sister.

In addition, *LDHA* was upregulated in Subject 1, potentially increasing glycolytic activity and lactate metabolism. This is particularly relevant, as TANGO2 loss is known to impair mitochondrial function [19], leading to episodes of acute metabolic crisis characterized by lactic acidosis and elevated serum lactate [9]. The *LDHA* upregulation may represent a compensatory shift toward anaerobic glycolysis in response to mitochondrial dysfunction, further contributing to the severity of TDD in Subject 1.

**Figure 12:**
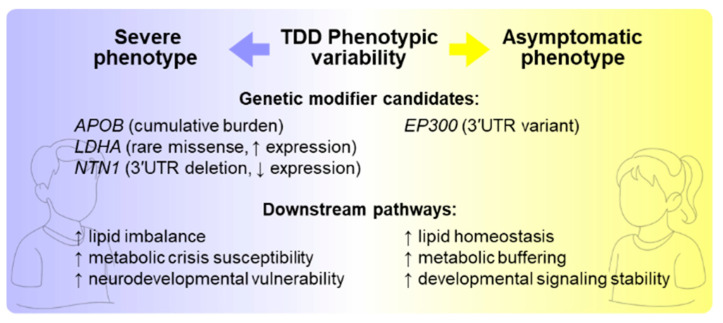
Modifier-driven mechanisms of phenotypic divergence in TANGO2 deficiency. Genetic modifier candidates identified in Subject 1 (*APOB*, *LDHA*, *NTN1*) may exacerbate pathways linked to lipid dysregulation, susceptibility to metabolic crises, and neurodevelopmental vulnerability, consistent with his severe clinical presentation. In contrast, Subject 2 carries a potentially protective 3′UTR variant in *EP300* and lacks the homozygous *APOB* burden, aligning with enhanced metabolic buffering, lipid homeostasis, and stabilized developmental signaling, consistent with an asymptomatic phenotype.

In summary, our analysis uncovered specific molecular mechanisms potentially modulating disease severity, identifying promising targets for further exploration. Our findings highlight that common polymorphisms can act as significant genetic modifiers in monogenic disorders, moving beyond single-gene causality toward a more comprehensive genotype–phenotype framework. Building on these findings, future experimental validations, such as in vitro functional assays of *APOB* and *VLDLR* variants, TANGO2 interactome studies, and investigations into the regulatory roles of *EP300* in autophagy and mitophagy, could be feasible and poised to deepen understanding of the molecular basis of phenotypic variability (Figure 12). Moreover, in the future, in vivo models incorporating these modifiers could be developed to clarify their impact on disease progression and metabolic resilience. Furthermore, expanding the collection of genomic and phenotypic data from other patients will be crucial to validate the proposed candidates and determine whether their effects are consistent across the TDD population.

## 5. Conclusions

Our integrated multi-omics analysis identified potential genetic modifiers that may underlie the striking phenotypic differences between the two siblings.

This study demonstrated that Systems biology approaches can extract meaningful insights from rare disease cohorts, successfully identifying genetic modifiers and mechanisms of disease heterogeneity from only two TDD patients. Importantly, this work shows that actionable biological insights can be obtained even from extremely limited patient cohorts when multiple computational strategies are applied. More broadly, this integrated framework provides a robust template for investigating phenotypic variability in other rare monogenic disorders, opening new avenues for precision medicine in rare disease populations.

## Figures and Tables

**Table 1:** Summary of clinical features in two siblings harboring identical *TANGO2* mutations.

Category	Subject 1 (Brother-Severe TDD Phenotype)	Subject 2 (Sister-Asymptomatic)
**Demographics**		
Ethnicity/Race	Non-Hispanic, Mixed (Italian + Indian)	Non-Hispanic, Mixed (Italian + Indian)
Sex	Male	Female
Age at TDD diagnosis (years)	2.5	1
Age of last follow-up	7.4	5.9
**Genetics**		
Chromosome Location	22q11	22q11
Variants	c.262C>T|c.338delG	c.262C>T|c.338delG
**Developmental Delays**		
Motor Delays	Severe motor delays (e.g., sitting at 8 months, rolling over 9 months, crawled at 18 months)	No significant delays reported
Speech Delay	Speech delay (limited vocabulary, difficulty forming full sentences)	No significant delays reported
**Motor Impairment**		
Muscle Weakness	Yes	No
Balance Issues	Yes	No
Head Tilt	Yes	No
**Cognitive/Intellectual Impairment**		
Approximate Intellectual Age (years)	2.5	Normal for age
History of Cognitive Delay	Cognitive/intellectual impairment	No
**Neurological Disorders**		
Intellectual disability	Yes	No
Abnormal EEG	No	No
Reaction to Loud Noises	Significant reaction to loud noises	No
**Cardiac & Metabolic Issues**		
Metabolic Crises	3	0
Rhabdomyolysis	Yes	No
Cardiac Arrest (Age)	1 (1.1 years)	No
Prolonged QTc	Yes	No
**Other Clinical Findings**		
Vision Problems	Yes	No
Hypothyroidism	Yes	No
History of Developmental Regression	No	No

**Table 2:** Rare gene variants with high predicted pathogenicity identified in the two siblings.

Gene Symbol	HGVSc	HGVSp	Existing Variation	Allele Frequency	CADD Score	pLI ^a^	Subject
*ACACB*	c.2295+1G>A		rs746447307	1.314 × 10^−5^	33	0	1
*ANKRD13B*					32	0	1
*CLN5*	c.525del	p.Trp175Ter	rs587780315	6.57 × 10^−6^	34	0	2
*CUX1*	c.3128C>T	p.Ser1043Leu	rs146486358	3.941 × 10^−4^	33	1	1–2
*DDX20*	c.1310C>T	p.Pro437Leu	rs199513956	3.88 × 10^−4^	34	0	1
*DDX56*	c.1318C>T	p.Gln440Ter			48	0	2
*GAL3ST4*	c.1357C>T	p.Arg453Cys	rs150667953	4.735 × 10^−4^	32	0	1–2
*GALNT9*	c.976G>A	p.Gly326Ser	rs1245240239		32	0	1
*GCA*	c.556C>T	p.Arg186Ter	rs141572054	0.002	40	0.04	1
*GJC2*	c.980T>A	p.Leu327Ter	rs891354098	6.765 × 10^−6^	34	0.46	2
*GOLIM4*	c.2005C>T	p.Arg669Ter	rs141565580	4.609 × 10^−5^	35	0	1–2
*GRM6*	c.1639C>T	p.Arg547Cys	rs182751201	0.003	33	0	2
*GXYLT1*	c.97G>T	p.Gly33Ter	rs1262821887	0.004	37	0	1–2
*KCNJ12*	c.236G>C	p.Arg79Pro	rs377320706	9.192 × 10^−5^	32	1	2
*KRT27*	c.847-1G>A		rs187944199	0.002	34	0	2
*KRT82*	c.139C>T	p.Arg47Ter	rs148453926	0.002	39	0	2
*LIFR*	c.2578C>T	p.Arg860Trp	rs146205670	0.001	32	0	1–2
*LIN7B*	c.27del	p.Leu10TrpfsTer84			32	0	1
*MKNK2*	c.758C>T	p.Ser253Leu	rs2016896796		34	0	2
*MUC19*	c.23324del	p.Gly7775GlufsTer?	rs748533284	8.546 × 10^−5^	34		2
*NLRP14*	c.322A>T	p.Lys108Ter	rs76274604	0.005	34	0	1–2
*NOC2L*			rs367769858	4.604 × 10^−5^	34	0	2
*OBSL1*	c.4732C>T	p.Gln1578Ter	rs116131367	0.003	45	0	2
*PABPC1*	c.1033G>T	p.Glu345Ter	rs142985461	0.002	45	1	1
*PAX7*	c.644G>A	p.Arg215His	rs200575057	4.731 × 10^−4^	33	0.98	2
*PCDHGA6*	c.1331A>G	p.Asp444Gly	rs752900319	6.571 × 10^−6^	30	0	2
*PIEZO1*					33	0	1–2
*PIK3C2G*	c.2627del	p.Ile876ThrfsTer23	rs765216023	1.315 × 10^−4^	34	0	2
*PLIN2*	c.214C>G	p.Leu72Val	rs149124591	0.004	34	0	2
*POLR2E*	c.496C>T	p.Arg166Ter	rs201275503	3.958 × 10^−5^	48	0	2
*PSD2*					34	0	2
*PSTPIP2*	c.642+1G>T				34	0	1
*RANBP3*	c.313G>A	p.Gly105Arg	rs749355669		31	1	2
*SCGN*	c.83-1del		rs60502981	0.009	34	0	2
*SCYL3*	c.1010G>A	p.Arg337Gln	rs143546688	0.002	33	0	1
*SLC2A8*	c.617C>T	p.Pro206Leu	rs372901354	3.284 × 10^−5^	30	0	1
*SORT1*	c.1108+1G>A				34	0.83	2
*SPNS1*	c.1550G>A	p.Arg517His	rs149610167	0.005	32	0.03	1–2
*SPTA1*	c.823_827del	p.Glu275HisfsTer14	rs1654503475		35	0	1–2
*SPTA1*	c.817_820del	p.Val273LeufsTer43	rs1654503795		33	0	1–2
*TANGO2*	c.262C>T	p.Arg88Ter	rs140115503	5.473 × 10^−5^	36	0	1–2
*TIE1*	c.1297G>A	p.Gly433Ser	rs767153303	1.314 × 10^−5^	32	0	2
*TMEM64*	c.767C>A	p.Pro256His	rs144669410	1.971 × 10^−5^	32	0	1–2
*TRPM8*	c.1423C>T	p.Arg475Cys	rs201940567	5.259 × 10^−4^	32	0	1–2
*ZNF208*	c.2266A>T	p.Lys756Ter	rs370724361	1.118 × 10^−4^	34	0	2
*ZNF256*	c.856C>T	p.Arg286Ter	rs748369479	6.574 × 10^−6^	33	0.28	2
*ZNF268*	c.2482C>T	p.Arg828Ter	rs200561453	0.001	35	0	1
*ZNF468*	c.766C>T	p.Arg256Ter	rs531943295	4.602 × 10^−5^	33	0	1

Gene variants were retained after filtering for a gnomAD allele frequency < 0.01 (or not annotated) and a CADD score ≥ 30. ^a^ The pLI score is retrieved from the Genome Aggregation Database (gnomAD).

**Table 3:** Core genes in the functional interaction (FI) network.

Gene Symbol	Edge Count	In-Degree	Out-Degree
*EP300*	115	24	91
*SP1*	85	72	13
*RPS27A*	84	62	22
*JUN*	77	32	45
*UBC*	73	72	1
*SRC*	71	61	10
*RAC1*	67	57	10

Seven highly connected genes were identified within the FI network generated from differentially expressed genes.

**Table 4:** Potentially impactful variants mapped to genes within the giant components of personalized integrated networks.

Gene Symbol	Giant Component	HGVSc	HGVSp	Existing Variation	Allele Frequency	CADD Score	pLI ^a^	Status ^b^	Subject ^c^
*ACAN*	2	c.5069G>A	p.Gly1690Glu	COSV61356763		18.91	1		1–2
*ALDH1A2*	2	c.1042G>A	p.Val348Ile	rs4646626	0.458	15.99	0.17	down	1–2
*APOA2*	1			rs3813628	0.289	17.98	0.35		1
*APOB*	1–2	c.8216C>T	p.Pro2739Leu	rs676210	0.230	27.5	0		1 †
*APOB*	1–2	c.1853C>T	p.Ala618Val	rs679899	0.401	26.7	0		1 †–2
*APOB*	1–2	c.293C>T	p.Thr98Ile	rs1367117	0.242	21.3	0		2
*ARPC5*	1–2	c.91G>A	p.Gly31Ser	rs371421898	1.31 × 10^−5^	21.4		up	1–2
*ARPC5*	1–2	c.-111T>C		rs2767305	0.527	18.88		up	1
*ASCC3*	1–2	c.436C>T	p.Leu146Phe	rs9390698	0.346	16.06	0	up	2
*ASCC3*	1–2	c.5984C>G	p.Ser1995Cys	rs240780	0.682	15.46	0	up	1 †–2 †
*ASCC3*	1–2	c.2080-24T>G		rs9403997	0.427	15.05	0	up	2
*BDNF*	1–2	c.-21-15778G>T		rs10835210	0.330	16.7	1	up	1–2
*BMP4*	2	c.-7-39A>G		rs2761880	0.836	16.86	1	down	1 †–2 †
*CDKN1B*	2			rs71064312	0.425	17.33	0.24		1–2
*CDKN1B*	2	c.326T>G	p.Val109Gly	rs2066827	0.370	16.88	0.24		1–2 †
*CHD3*	1–2	c.100+209C>T				22.2	1	down	1
*CHD3*	1–2	c.100+47G>C		rs2279620	0.106	15.69	1	down	2
*CYBA*	1–2	c.214T>C	p.Tyr72His	rs4673	0.653	21.3	0	up	1 †–2 †
*DNM1*	1–2	c.2535-1654G>A		rs2267958	0.385	21.9	1	down	1–2
*DOCK2*	2	c.321+87C>T		rs12656761	0.199	22.4	1	up	1–2
*DOCK3*	1–2	c.1918-3T>C		rs1480361	0.991	15.58	1	up	1 †–2 †
*EP300*	2	c.*13_*15del		rs35508493	0.246	15.8	1		2
*EVC*	2	c.1727G>A	p.Arg576Gln	rs1383180	0.321	25.4	0	down	1–2
*EVC*	2	c.772T>C	p.Tyr258His	rs6414624	0.729	17.5	0	down	1 †–2 †
*EVC*	2	c.969T>C	p.Asn323%3D	rs4688963	0.412	16.14	0	down	1 †–2 †
*EVC*	2	c.221A>C	p.Gln74Pro	rs2291157	0.083	15.84	0	down	1–2
*FBLN1*	2	c.422A>G	p.Gln141Arg	rs136730	0.999	16.38	0.95	down	1 †–2 †
*FOXF1*	2			rs77626209	0.677	16.1	0.43	down	1 †–2 †
*GLI1*	2			rs512490	0.887	16.87	0		1 †–2 †
*GRIA4*	1	c.2295-5718G>A		rs632549	0.999	16.05	16.1	down	1 †–2 †
*GRIA4*	1	c.1269+1231_1269+1232del		rs60005308	0.009	15.95	16.1	down	1
*GRIA4*	1	c.2544+250C>T		rs593130	0.647	15.39	16.1	down	2 †
*GRIN2D*	1	c.*132G>T		rs8111684	0.623	18.63		down	1 †
*HGF*	1–2	c.1541+159T>G		rs2074725	0.747	21	1		1 †–2 †
*JPH2*	2	c.1186G>A	p.Ala396Thr	rs3810510	0.201	21.3	0	up	1–2
*KCNH2*	2	c.2690A>C	p.Lys897Thr	rs1805123	0.210	21.7	0.07		1
*KCNH2*	2	c.-43_-39dup		rs747534042	1.865 × 10^−4^	21	0.07		2
*KCNH2*	2	c.77-134dup		rs5888422	1.000	16.32	0.07		1 †–2 †
*LAMB1*	2	c.2441G>T	p.Gly814Val	rs556203005	1.971 × 10^−5^	27.2	0		1–2
*LDHA*	1	c.439G>T	p.Ala147Ser	rs116841148	0.001	23.8	0.77	up	1
*LIPC*	1–2	c.1068C>A	p.Phe356Leu	rs3829462	0.946	18.28	0	down	1 †–2 †
*LTBP4*	2	c.4298A>T	p.Tyr1433Phe	rs35809725	0.009	22.6	0	down	1–2
*LTBP4*	2	c.3221C>T	p.Thr1074Met	rs10880	0.392	18.86	0	down	1–2
*LYN*	2	c.-82G>A		rs1050855	0.505	16.76	1	up	2
*MDM2*	1	c.*1982G>A				21.5	1	up	1
*MDM2*	1	c.-94A>G		rs937283	0.363	17.85	1	up	1
*MECOM*	1–2	c.376-2930A>T		rs1420476	0.963	15.34	1	up	1 †–2 †
*MSX2*	1–2	c.386T>C	p.Met129Thr	rs4242182	0.779	21.7	0.01	down	1 †–2 †
*MYO6*	1	c.2077+96T>A		rs9443195	0.338	15.86	0.97	up	1 †–2
*NGF*	1	c.104C>T	p.Ala35Val	rs6330	0.356	21.8	0.77		1
*NTN1*	1	c.*30_*33del		rs762914852	2.56 × 10^−4^	16.28	1	down	1
*PDGFRB*	1–2	c.946G>A	p.Val316Met	rs41287112	0.003	17.86	0.94	down	1–2
*PIK3R1*	1–2	c.917-3329A>G		rs1862162	0.222	15.88	1		1–2
*PKD2*	2	c.83G>C	p.Arg28Pro	rs1805044	0.234	20.4	0	up	1–2
*PLOD2*	2	c.338+4G>A		rs4681297	0.777	16.55	0	up	1 †–2
*PRKACA*	2	c.47-270T>C		rs35374974	0.213	15.78	1		2
*PSMC3IP*	2	c.338-15C>G		rs2292752	0.574	17.58	0	up	1–2 †
*PTPN22*	1–2	c.1858T>C	p.Trp620Arg	rs2476601	0.934	15.54	0	up	1 †–2 †
*RET*	1–2	c.3187+198T>A		rs2075913	0.757	22.1	1		1–2
*RET*	1–2	c.2712C>G	p.Ser904%3D	rs1800863	0.173	17.21	1		2
*RET*	1–2	c.73+53G>A		rs12267460	0.375	16.18	1		1–2
*RET*	1–2	c.3187+47T>C		rs2075912	0.831	15.58	1		1 †–2 †
*ROR2*	2	c.2455G>A	p.Val819Ile	rs10761129	0.715	15.69	0.35	down	2
*SPINT2*	1–2	c.337+42A>G		rs8104823	0.135	15.15	0	up	1–2
*TCF12*	1–2	c.222+8793G>A		rs2951904	0.570	17.32	0.02		1–2
*TIAM1*	1–2	c.740G>T	p.Gly247Val	rs2070417	0.116	23.4	0.99	up	1 †–2
*TIAM1*	1–2	c.739G>A	p.Gly247Arg	rs2070418	0.116	19.54	0.99	up	1 †–2
*TNC*	2	c.5341G>A	p.Ala1781Thr	rs2274750	0.056	25.8	0	down	2
*TNC*	2	c.2049G>A	p.Glu683%3D	rs2274836	0.440	17.7	0	down	2
*TNC*	2	c.6022G>C	p.Glu2008Gln	rs13321	0.708	17.31	0	down	1 †–2 †
*TNC*	2	c.5029A>T	p.Ile1677Leu	rs2104772	0.471	15.25	0	down	1–2
*VLDLR*	1–2	c.-69A>G		rs12379259	0.811	18.51	0	up	1–2
*VLDLR*	1–2	c.-56C>T		rs34881325	0.318	17.4	0	up	1
*VLDLR*	1–2	c.82+7G>A		rs2219143	0.315	16.9	0	up	1
*VLDLR*	1–2	c.-43_-35del		rs71329437	0.329	16.53	0	up	1

Variants classified as variants of uncertain significance (VUS) or as predicted pathogenic (CADD ≥ 15) were listed for Subject 1 and Subject 2. ^a^ The pLI score is retrieved from the Genome Aggregation Database (gnomAD). ^b^ Gene expression changes detected through differential expression analysis: up indicates upregulation in Subject 1, down indicates downregulation in Subject 1, and an empty cell indicates no significant change in gene expression. ^c^ The † symbol next to the Subject ID indicates homozygosity for the variant, absence of the † symbol indicates heterozygosity.

## Data Availability

The raw data supporting the conclusions of this article are available at https://doi.org/10.5281/zenodo.16887326.

## Supplementary Materials

The following supporting information can be downloaded at: https://www.mdpi.com/article/10.3390/biom15121742/s1. Table S1: Functional enrichment analysis of genes in the giant component of the PPI network; Table S2: Differentially expressed genes (DEGs) identified by univariate and multivariate analyses; Table S3: Human Phenotype Ontology (HPO) terms linked to TANGO2 deficiency disorder; Table S4. Genes included in the TANGO2 deficiency disorder (TDD) virtual panel; Table S5: Potentially impactful variants mapped to genes within the personalized integrated networks; Table S6: Functional enrichment analysis of genes in subject-specific subnetworks; Figure S1: Histogram of the CADD score distribution for all variants; Figure S2. Giant components of subject-specific PPI networks filtered for rare variants; Figure S3: Exploratory analysis of the matrix counts; Figure S4: Gene-wise dispersion estimation; Figure S5: sPLS-DA analysis; Figure S6: Heatmap of the 1000 DEGs obtained by the intersection of DESeq2 and sPLS-DA/RFE; Figure S7 Disease-phenotype network for TANGO2 deficiency disorder (TDD).

## Abbreviations

The following abbreviations are used in this manuscript:

Acyl-CoA	acyl-coenzyme A
AF	allele frequency
CADD	Combined Annotation Dependent Depletion
CPM	counts per million
DGE	Differential Gene Expression
DEGs	differentially expressed genes
ER	endoplasmatic reticulum
FDR	false discovery rate
FI	functional interaction
gnomAD	Genome Aggregation Database
GO	Gene Ontology
GSEA	gene set enrichment analysis
HPO	Human Phenotype Ontology
IMEx	International Molecular Exchange
LD	lipid droplets
ORA	over representation analysis
PCA	Principal Components Analysis
PPI	protein–protein interaction
RFE	Recursive Feature Elimination
ROS	reactive oxygen species
sPLS-DA	Sparse Partial Least Squares Discriminant Analysis
TANGO2	Transport and Golgi Organization 2 homolog
TDD	TANGO2 deficiency disorder
VCF	Variant calling format
VEP	Variant Effect Predictor
VUS	variant of unknown significance
WES	whole exome sequencing
