# Supplementary material for: Systems-Level Integration of Multi-Omics Identifies Genetic Modifiers of TANGO2 Deficiency Disorder
Source: Biomolecules. 2025 Dec 16;15(12):1742. doi: 10.3390/biom15121742 (PMC12730736; doi:10.3390/biom15121742)
Supplement: Supplementary file 1 [file biomolecules-15-01742-s001.zip › Supplementary_Materials_1.pdf]

Systems-level integration of multi-omics identifies genetic modifiers of TANGO2 deficiency disorder

Manuel Airoldi[1†], Heather Bondi[1†], Veronica Remori[1], Silvia Carestiato[2], Giovanni Battista Ferrero[3,4], Alfredo Brusco[2,5], Mauro Fasano[1,6*]

[1] Department of Science and High Technology, University of Insubria, Como, Italy
[2] Department of Neuroscience "Rita Levi Montalcini", University of Turin, Turin, Italy
[3] Medical Genetics Unit and Thalassemia Center, San Luigi University Hospital, Orbassano, Italy
[4] Department of Clinical and Biological Sciences, University of Turin, Turin, Italy
[5] Medical Genetics Unit, Città della Salute e della Scienza University Hospital, Turin, Italy
[6] Neuroscience Research Center, University of Insubria, Busto Arsizio, Italy
* Correspondence: mauro.fasano@uninsubria.it
† These authors contributed equally to this work and share first authorship

Supplementary figures

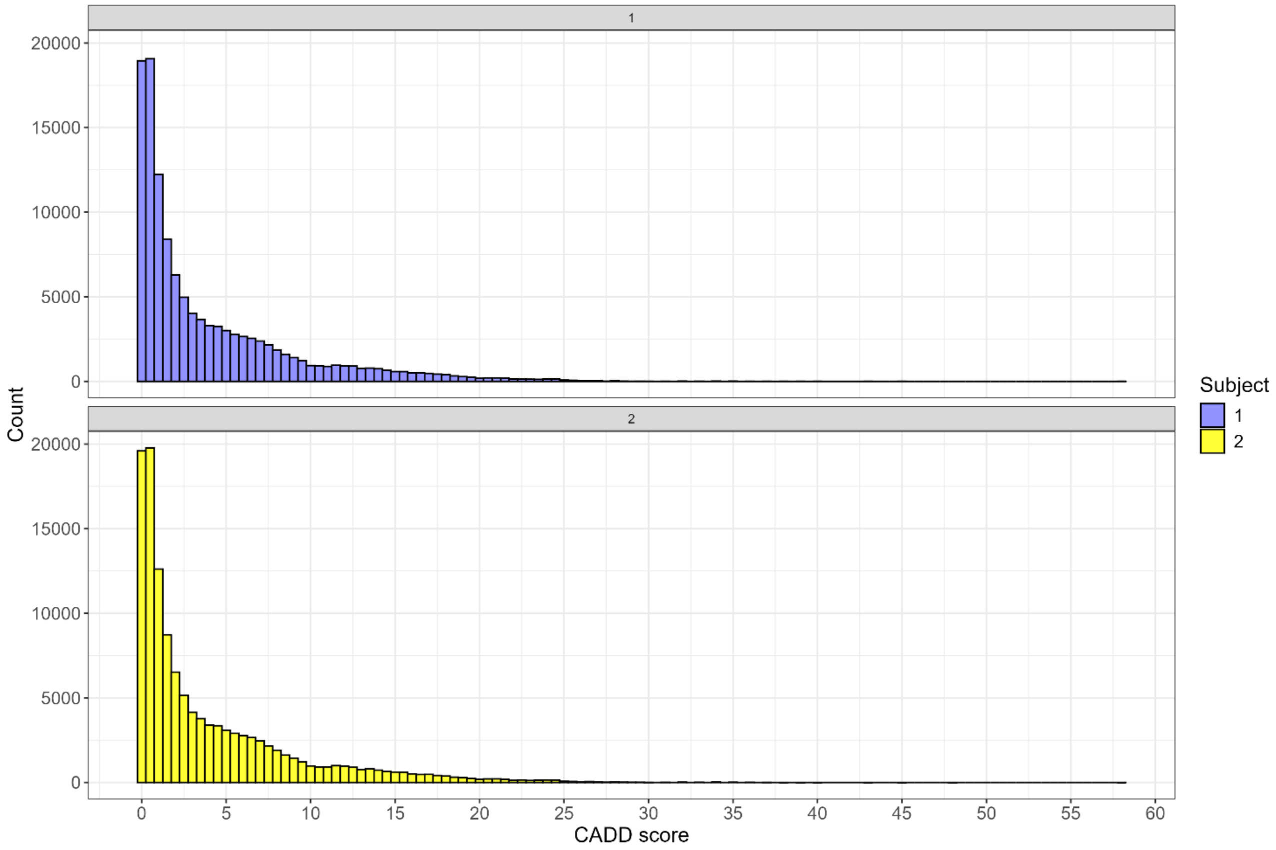

Figure S1. Histogram of the distribution of CADD score for all variants. Blue = subject 1, yellow = subject 2.

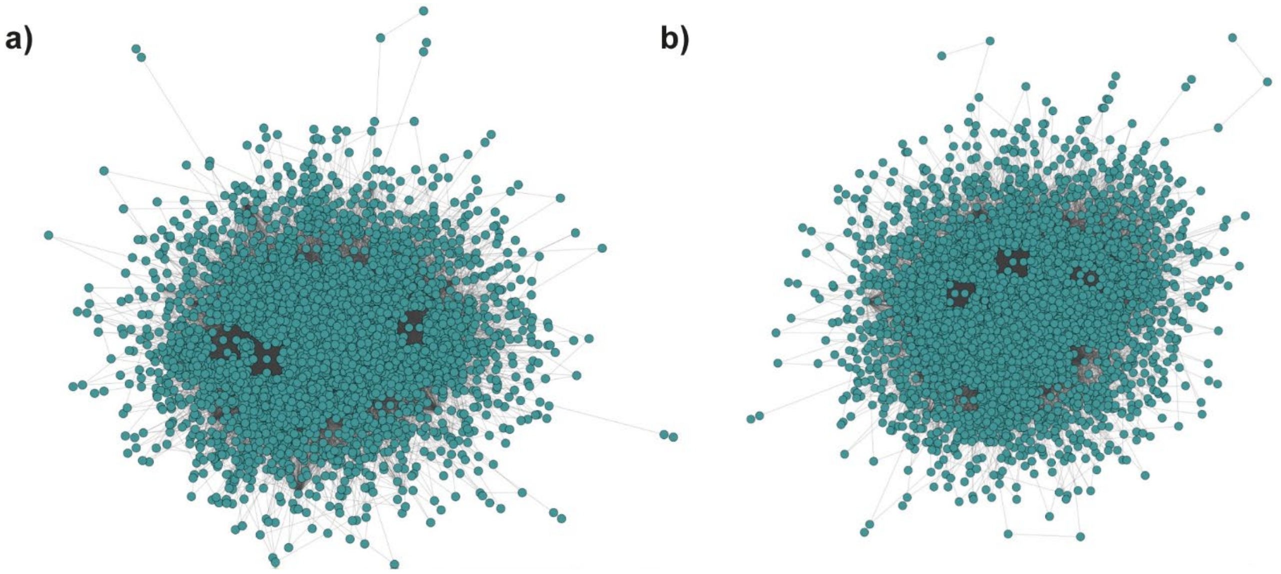

Figure S2.**Giant components of subject-specific PPI networks filtered for rare variants.** Protein–protein interaction (PPI) networks were built from ES-derived genes carrying at least one variant, then filtered to retain only those with rare variants (AF < 0.01 or not annotated in GnomAD). Panel (**a**) shows the giant component for Subject 1; panel (**b**) for Subject 2.

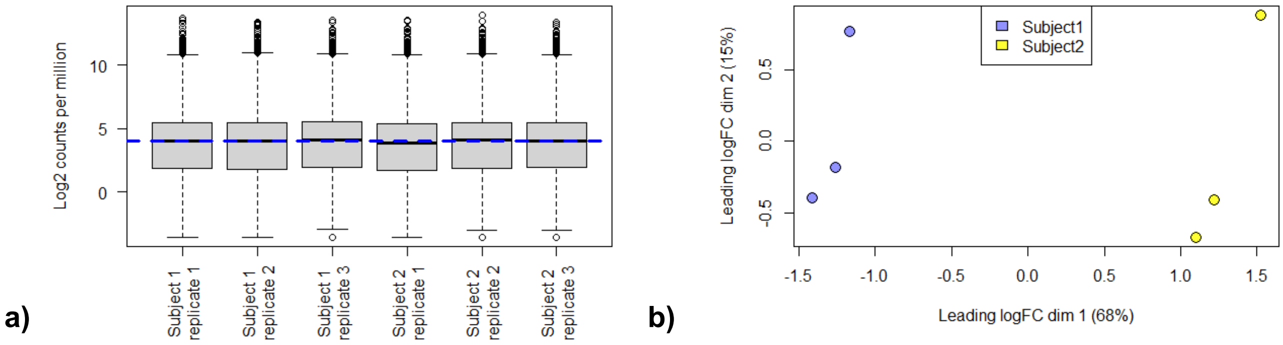

Figure S3. Explorative analysis of the matrix counts. **a)** Boxplot of log_2CPM of all replicates. The blue dashed line represents the median of the CPMs. All boxplots are similar to each other. **b)** Principal Component Analysis. X axis represents the first dimension, Y axis represents the second dimension.

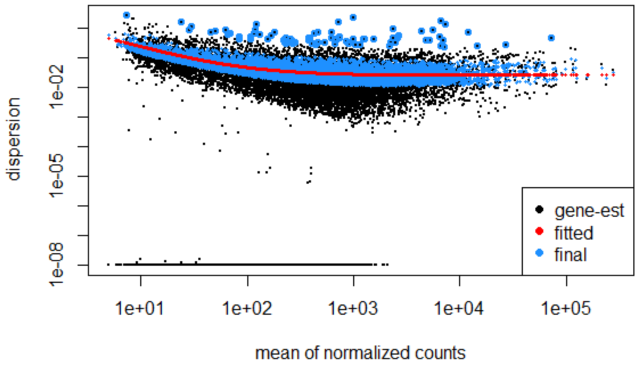

Figure S4. Fitting of a curve to gene-wise dispersion estimation. The fitted curve is shown as a red line, which plots the estimate for the expected dispersion value for genes of a given expression strength. Each black dot is a gene with an associated mean expression level and maximum likelihood estimation of the dispersion. The blue dots are the genes modeled by DESeq2.

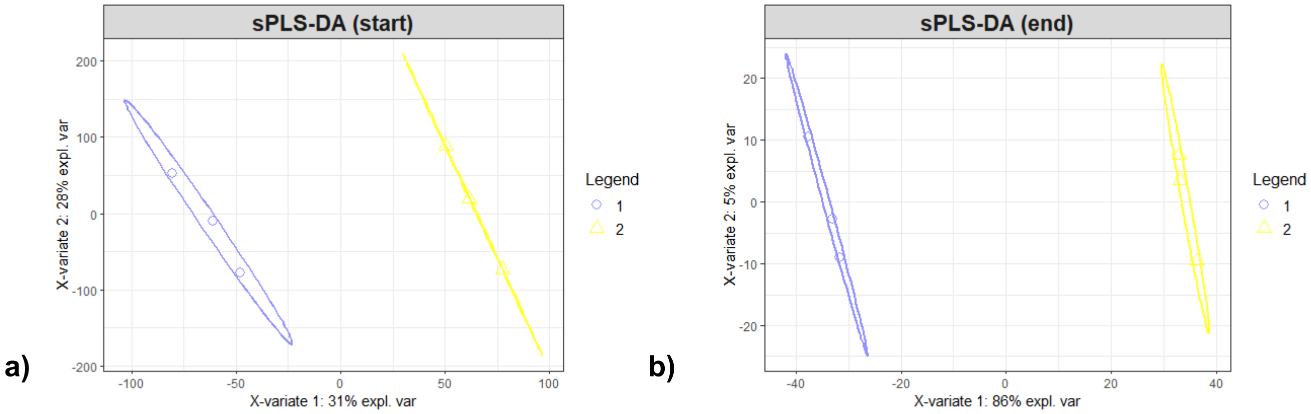

Figure S5. sPLS-DA analysis. **a)** sPLS-DA performed on the initial 16403 × 6 matrix. X axis = component 1 (it explains 31% of variance). Y axis = component 2 (it explains 28% of variance). **b)** sPLS-DA performed on the final matrix after RFE. X axis = component 1 (it explains 86% of variance). Y axis = component 2 (it explains 5% of variance).

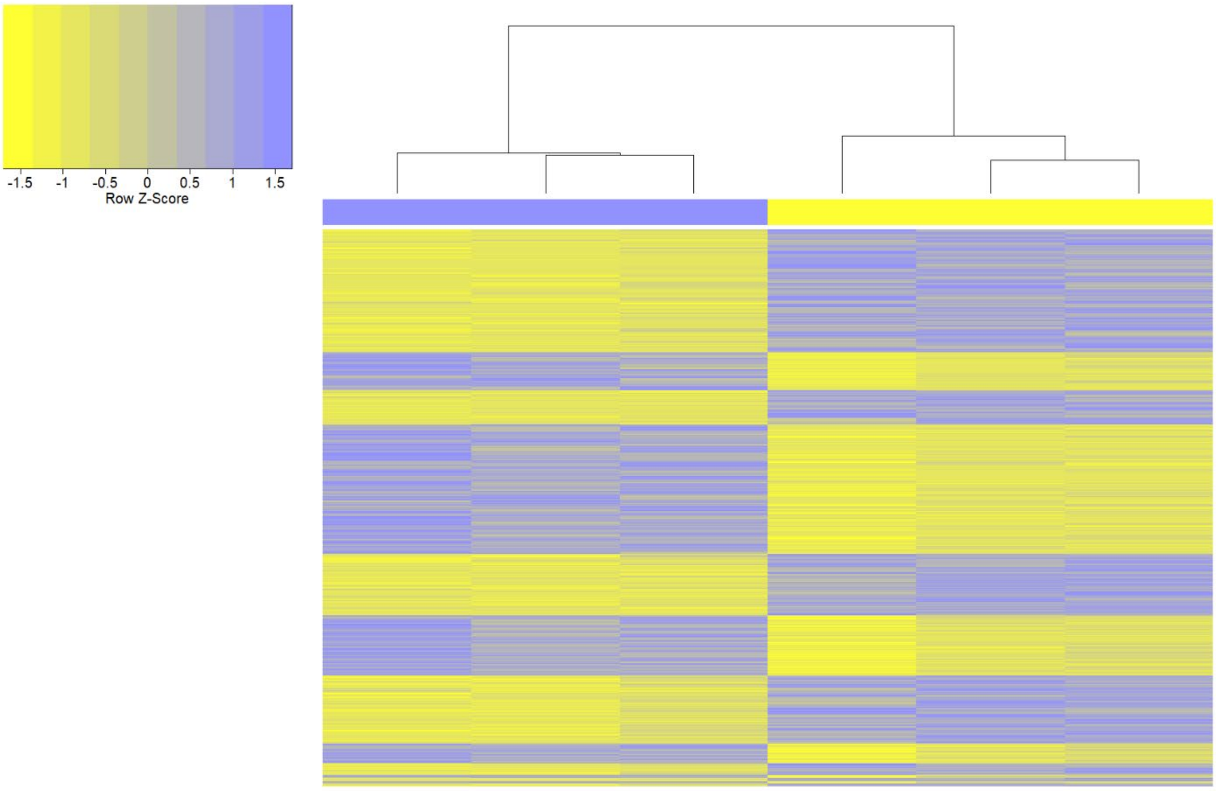

Figure S6. Heatmap of the 1000 DEGs obtained by the intersection of the two approaches. Colors span from yellow (down-regulated) to blue (up-regulated). The bar on the top indicates the group: blue = subject 1, and yellow = subject 2.

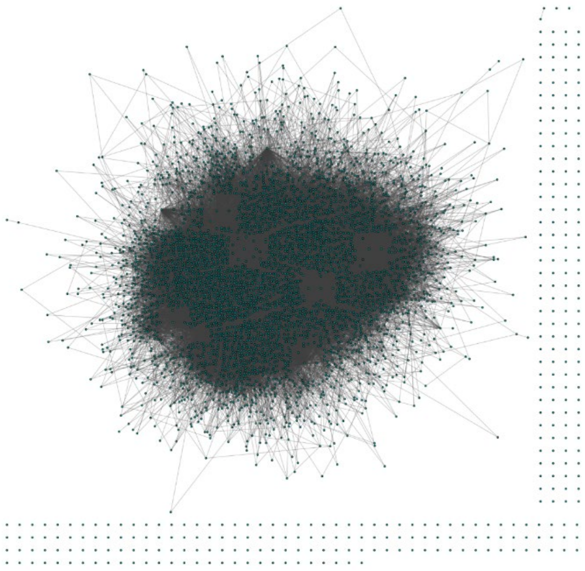

Figure S7. Disease-phenotype network for TANGO2 deficiency disorder (TDD). Protein–protein interaction (PPI) network generated from 4,599 genes derived from the Human Phenotype Ontology (HPO) and associated with TDD-related phenotypes (OMIM: #616878). The network represents functional connections among phenotype-associated genes
